# Effect of a tumour-derived lipid-mobilising factor on glucose and lipid metabolism *in vivo*

**DOI:** 10.1038/sj.bjc.6600493

**Published:** 2002-08-27

**Authors:** S T Russell, M J Tisdale

**Affiliations:** Pharmaceutical Sciences Research Institute, Aston University, Birmingham B4 7ET, UK

**Keywords:** cachexia, lipid mobilising factor, glucose and lipid metabolism

## Abstract

Treatment of ex-breeder male NMRI mice with lipid mobilising factor isolated from the urine of cachectic cancer patients, caused a significant increase in glucose oxidation to CO_2,_ compared with control mice receiving phosphate buffered saline. Glucose utilisation by various tissues was determined by the 2-deoxyglucose tracer technique and shown to be elevated in brain, heart, brown adipose tissue and gastrocnemius muscle. The tissue glucose metabolic rate was increased almost three-fold in brain, accounting for the ability of lipid mobilising factor to decrease blood glucose levels. Lipid mobilising factor also increased overall lipid oxidation, as determined by the production of ^14^CO_2_ from [^14^C carboxy] triolein, being 67% greater than phosphate buffered saline controls over a 24 h period. There was a significant increase in [^14^C] lipid accumulation in plasma, liver and white and brown adipose tissue after administration of lipid mobilising factor. These results suggest that changes in carbohydrate metabolism and loss of adipose tissue, together with an increased whole body fatty acid oxidation in cachectic cancer patients, may arise from tumour production of lipid mobilising factor.

*British Journal of Cancer* (2002) **87**, 580–584. doi:10.1038/sj.bjc.6600493 www.bjcancer.com

© 2002 Cancer Research UK

## 

Loss of whole body fat is a prominent feature of cancer cachexia, with losses of up to 85% being reported (Fearon, 1992) in lung cancer patients, who had lost 30% of their pre-illness stable weight. Most studies suggest that loss of fat arises as a result of an increased lipolysis, together with increased whole body fatty acid oxidation. Thus increased plasma concentrations of glycerol, free fatty acids (FFA) and triglycerides were observed in a heterogeneous group of cancer patients with an average loss of 13% of their original body weight (Legaspi *et al*, 1987). Basal fatty acid turnover was elevated by 25% above that for controls, and was found to be similar to the rate observed for patients with severe burns. Lipolysis was increased by 40% in patients in whom complete triglyceride hydrolysis without re-esterification was observed, and there was a 20% increase in fatty acid oxidation.

Upregulation of catabolism, rather than defects in anabolism, appear to be most important in the loss of lipid from human adipose tissue in cancer cachexia. Thus Thompson *et al* (1993) have shown a two-fold increase in the relative level of mRNA for triglyceride lipase, while the relative levels of mRNA for lipoprotein lipase (LPL) and fatty acid synthase were not significantly different between cancer patients and controls. This suggests that cytokines such as tumour necrosis factor-α (TNF-α), interleukin-1 (IL-1), interleukin-6 (IL-6), interferon-γ (IFN-γ) and leukaemia-inhibitory factor (LIF), which have been proposed to decrease adipose tissue mass by decreasing synthesis of triglycerides through inhibition of LPL (Berg *et al*, 1994) probably play a minor role in the loss of lipid from human adipose tissue. It therefore seems more likely that lipid mobilisation in cancer cachexia can be attributed to tumour catabolic factors, such as lipid mobilising factor (LMF), which acts directly on adipose tissue with the release of FFA and glycerol in a manner similar to that of lipolytic hormones (Beck and Tisdale, 1987). LMF has been purified from the urine of patients with cancer cachexia using a combination of ion exchange, exclusion and hydrophobic interaction chromatographies (Todorov *et al*, 1998). Unlike polypeptide hormones stimulating lipolysis which are basic, LMF is acidic and showed homology in amino acid sequence, electrophoretic mobility and immunoreactivity with the plasma protein Zn-α_2_-glycoprotein (ZAG). Both LMF and ZAG stimulate adenylate cyclase in adipocyte plasma membranes in a GTP-dependent process (Hirai *et al*, 1998). This suggests the two factors are similar, although they may differ in glycosylation. Administration of LMF to obese mice produced a specific loss of carcass lipid, without a change in body water or nonfat mass. Oxygen uptake by interscapular brown adipose tissue (BAT) was increased three-fold, providing evidence for increased lipid utilisation. Despite fat mobilisation there was a decrease in blood glucose levels. Hypoglycaemia occurs in mice bearing a cachexia-inducing tumour (MAC16), although the mechanism by which this occurs is not known (Mcdevitt and Tisdale, 1992).

The purpose of the present study was to investigate the effect of LMF on glucose and lipid metabolism *in vivo* in order to quantitate the effect on energy utilisation.

## MATERIALS AND METHODS

### Animals

Ex-breeder male NMRI mice (35–40 g) were obtained from our own breeding colony and were fed a rat and mouse diet (Special Diet Services, Witham, Essex, UK). All animal experiments followed a strict protocol, agreed with the British Home Office, and the ethical guidelines that were followed meet the standards required by the UKCCCR guidelines (Workman *et al*, 1998).

### Radiochemicals

D-[U-^14^C]glucose (sp. act. 11.0 GBq mmol^−1^), 2-deoxy-D-[2,6-^3^H]glucose ([^3^H]2DG; sp. act. 1.63 TBq mmol^−1^) and 2-deoxy-D-[1-^14^C]glucose ([^14^C]2DG; sp. act. 2.072 MBq mmol^−1^), were purchased from Amersham Lifesciences, (Bucks, UK). [Carboxy ^14^C] Triolein (sp. act. 3.8 GBq mmol^−1^) was from New England Nuclear (Southampton, UK).

### Purification of LMF

**Figure 1:**
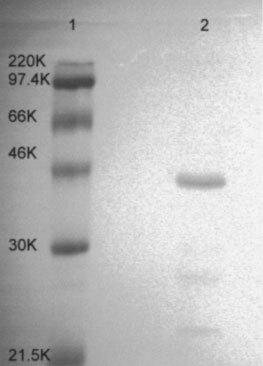
Electrophoretic separation of proteins on a 12% SDS polyacrylamide gel. Lane 1 molecular weight markers; Lane 2 LMF (5 μg protein) eluted from Resource-Iso column at 0.6 M (NH4)_2_SO_4_.

LMF was purified from the urine of weight losing patients with pancreatic cancer using a combination of batch extraction on DEAE cellulose and hydrophobic interaction chromatography as previously described (Todorov *et al*, 1998). Urine was centrifuged at 3000 g for 10 min to remove particulate material and diluted with 4 vol 10 mM Tris. HCl, pH 8.0. DEAE cellulose (10 g l^−1^ of original urine) was then added and the mixture was stirred for 2 h at 4°C. The LMF–DEAE cellulose complex was isolated by low speed centrifugation, and LMF eluted with 0.5 M NaCl in 10 mM Tris. HCl, pH 8.0. Bioactivity was monitored by the release of glycerol from freshly isolated epididymal adipocytes (Beck and Tisdale, 1987). The eluate was equilibrated against PBS and concentrated to 1 ml before further purification using a Resource-Iso HPLC column (Pharmacia Biotech, St Albans, Herts, UK) employing a decreasing (NH_4_)_2_SO_4_ concentration from 1.5 M. Active fractions containing LMF eluted at 0.6 M (NH_4_)_2_SO_4_ and were desalted before use by washing five times against PBS using an Amicon filtration cell. LMF eluted mainly as a single protein band of M_r_ 43 000, as determined by Coomassie blue staining of a 12% SDS polyacrylamide gel (Figure 1

).

### Treatment of animals

LMF (8 μg in 100 μl PBS) was administered b.d. by i.v. administation into the tail vein of ex-breeder male NMRI mice. This dose was previously shown (Hirai *et al*, 1998) to be effective in lipid depletion *in vivo*. Control animals received PBS alone. This was repeated up to 48 h before the effect on glucose and lipid utilisation was measured.

### Production of ^14^CO_2_ from D-[U-^14^C] glucose

Animals were injected i.p. with 50 μCi kg^−1^ of D-[U-^14^C] glucose in 200 μl of 0.9% NaCl and placed in airtight metabolic cages into which air was pumped through solid CaCO_3_ to absorb any CO_2_. Metabolically produced ^14^CO_2_ was trapped in glass test tubes containing 20 ml of a mixture of ethanolamine : ethoxyethanol (1 : 4). At specific time intervals (0.5, 1, 2, 4 and 8 h) 0.5 ml aliquots were taken and mixed with 10 ml Optiphase Hi-safe II (Fisher Chemicals, Leics, UK), and the radioactivity was measured in a Packard Tri-Carb 2000 CA scintillation analyser.

### Glucose utilisation

Glucose utilisation by various tissues after LMF administration was determined by the 2-deoxyglucose (2DG) tracer technique (Mesazaros *et al*, 1987a,b), with starvation overnight and throughout the experiment, but with *ad libitum* water. After the overnight starvation mice were injected i.v. with 50 μCi kg^−1^ [^3^H]2DG in 200 μl 0.9% NaCl and to determine the retention of 2-deoxyglucose-6-phosphate by the different tissues, a second i.v. injection of 5 μCi kg^−1^ of [^14^C]2DG was administered 35 min after the unjection of the tritiated deoxyglucose. The accumulation of phosphorylated metabolites of 2DG was measured in selected tissues 60 min after the injection of [^3^H]2DG. Mice were killed by cervical dislocation and blood removed from the heart for glucose measurement by the hexokinase-glucose 6-phosphate dehydrogenase enzymatic assay (Sigma-Aldrich Co. Ltd., Dorset, UK). The concentration of radioactivity in the blood was determined on deproteinised neutralised samples with a dual ^3^H/^14^C analyser. Tissues were homogenised in ice-cold 0.5 N perchloric acid at the rate of 0.4 ml per 100 mg tissue wet weight using a Camlab 563C homogeniser (speed 8) fitted with a teflon pestle. The homogenate was centrifuged for 15 min at 3000 r.p.m. and the supernatant was neutralised to pH 7 with 10% w v^−1^ potassium hydroxide, and the radioactivity was determined after the removal of the insoluble potassium perchlorate. This gave the total radioactivity of 2DG and its metabolites present in the tissue. 2DGP was removed from the neutral extract by precipitation with zinc sulphate/barium hydroxide and the difference between the total radioactivity of the neutral extract and that after removal of 2DGP represented the 2DGP content of the tissue.

Glucose utilisation was calculated from the equation (Meszaros *et al*, 1987a):


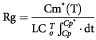


where Rg is the tissue glucose metabolic rate (nmol min^−1^g^−1^), Cm* (T) is the concentration of phosphorylated metabolites of 2DG in the tissue (d.p.m. g^−1^) at t=60 min, Cp is the blood glucose (nmol ml^−1^), Cp* is the concentration of [^3^H]2DG in the blood (d.p.m. ml^−1^) and LC (lumped constant) is a dimensionless correction factor for discrimination against 2DG in glucose metabolic pathways. This was determined to be 0.46 in NMRI mice (Mahony and Tisdale, 1990) using the method of Ferre *et al* (1985).

### Lipid oxidation and accumulation

The absorption, accumulation and oxidation of an oral dose of triolein was determined using the method of Oller do Nascimento and Williamson (1986). [Carboxy^14^C] triolein (0.33 μCi in 100 μl normal saline) was administered by intragastric intubation to NMRI mice previously administered either LMF or PBS. Immediately after administration animals were placed in airtight metabolic cages and expired ^14^CO_2_ was collected over a 24 h period as described above. At 5 and 24 h some of the animals were anaesthetised and blood was collected by cardiac puncture. The complete gastrointestinal tract was removed and homogenised in 5 ml of 3% perchloric acid. Lipids were extracted from organs and blood by the method of Stansbie *et al* (1976). The extracted fatty acids were dissolved in Optiphase Hi-safe II scintillation fluid and the radioactivity determined as above. Triolein absorption was calculated by subtracting the total gastrointestinal tract radioactivity from that administered.

### Statistical analysis

Results are expressed as mean±s.e.mean. Differences were determined by one-way Analysis of Variance (ANOVA) followed by Tukey–Kramer multiple comparison test. *P* values less than 0.05 were considered statistically significant.

## RESULTS

**Figure 2:**
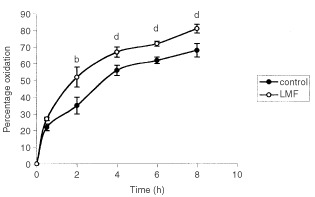
Production of ^14^CO_2_ from D-[U-^14^C] glucose by NMRI mice administered either LMF for 48 h or PBS as described in Materials and Methods. There were 12 mice in each group. Differences from control are indicated as either ^b^*P*<0.01 or ^d^*P*<0.001.

The effect of LMF administration for 48 h on ^14^CO_2_ production from D-[U-^14^C] glucose is shown in Figure 2

. This does not strictly measure glucose oxidation, since recycling of label and transfer to substrates such as lipids must also be considered, since they may contribute to the ^14^CO_2_ produced. In both groups between 70 and 80% of the administered radiolabel was metabolised to ^14^CO_2_ during an 8 h period. However, there was an increase in ^14^CO_2_ production from mice administered LMF, which became significant after 2 h, and remained above that of mice administered PBS alone over the 8 h experimental period.

**Figure 3:**
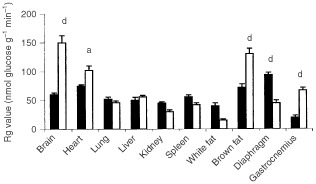
Tissue glucose metabolic rate (Rg) in mice administered either PBS (closed box) or LMF (open box) as described in Materials and Methods. There were 12 mice in each group. Differences from control are indicated as either ^a^*P*<0.05 or ^d^*P*<0.001.

In order to determine individual organ glucose utilisation after LMF, the 2DG tracer method was used (Meszaros *et al*, 1987a,b). The transport, cellular uptake and phosphorylation by hexokinase of this analogue correlate with those of glucose, but because 2DGP cannot readily be metabolised further, it can be detected in tissues containing little glucose-6-phosphatase, such as brain and muscle (Lackner *et al*, 1984; Jenkins *et al*, 1986). The tissue glucose metabolic rate (Rg) of LMF and PBS-treated mice is shown in Figure 3

**Figure 4:**
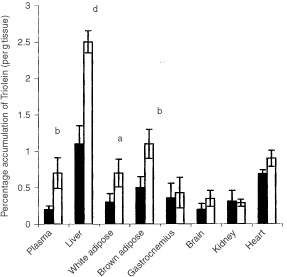
Tissue [^14^C] lipid accumulation (percentage absorbed dose per g tissue over a 6 h period) from [^14^C-carboxy] triolein administered enterally by gastric intubation as described in Materials and Methods after administration of either PBS (closed box) or LMF (open box). There were 12 mice in each group. Differences from control are indicated as either ^a^*P*<0.05, ^b^*P*<0.01 or ^d^*P*<0.001.

. Treatment with LMF caused a significant increase in Rg in brain, heart, brown adipose tissue (BAT) and gastrocnemius muscle, but a decrease in Rg in white adipose tissue (WAT) and diaphragm. The decrease in Rg in WAT correlates with an increased lipid utilisation (Figure 4

), although the reason for the difference between the two muscle types is not known. Since the brain is the main utilizer of glucose, the almost three-fold increase in Rg value observed in the presence of LMF (Figure 3) would account for the previously reported (Hirai *et al*, 1998) ability of LMF to decrease blood glucose levels.

The primary effect of LMF is on lipid mobilisation and utilisation through an increased rate of lipolysis (Hirai *et al*, 1998), and through stimulation of increased expression of mRNA for uncoupling protein 1 (UCP1) in BAT and uncoupling protein 2 (UCP2) in BAT, skeletal muscle and liver (Bing *et al*, 2002). The effect of LMF on the ability of mice to deal with administered lipid was investigated by intragastric intubation with [^14^C carboxy] triolein 48 h after treatment with either LMF or PBS and the absorption into various organs was monitored over a 6 h period (Figure 4). There was a significant increase in [^14^C] lipid accumulation in plasma, liver, WAT and BAT of LMF-treated mice compared with PBS controls, but no difference in gastrocnemius muscle, brain, kidney or heart. This distribution correlates with what would be expected from a decrease in lipid in plasma and WAT, and an increase in lipid in liver (Bing *et al*, 2002).

**Figure 5:**
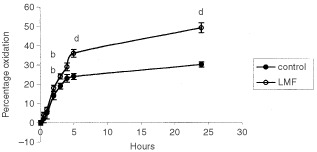
Production of ^14^CO_2_ from [^14^C carboxy] triolein after administration of either LMF or PBS as described in Materials and Methods. There were 12 mice in each group. Differences from control are indicated as either ^b^*P*<0.01 or ^d^*P*<0.001.

The rate of oxidation of [^14^C carboxy] triolein to ^14^CO_2_ for LMF and PBS-treated mice is shown in Figure 5

. Lipid oxidation increased exponentially during the first 5 h after administration of triolein in both groups and then levelled off up to 24 h. Animals administered LMF showed an increased rate of oxidation of [^14^C carboxy] triolein during the initial phase and overall lipid oxidation was 67% greater during the 24 h period. This result confirms the ability of LMF to increase lipid utilization *in vivo*.

## DISCUSSION

Loss of fat occurs when the metabolic demands on an organism are high, since fat constitutes 90% of the adult fuel reserves. Mobilisation of fat in cancer cachexia provides a fuel source for the host when the metabolic demand is high. Increased glucose utilisation by the tumour (Mulligan and Tisdale, 1991) results in an increased lactate production, resulting in an increased operation of the Cori cycle (Holroyde *et al*, 1975), which consumes 6 moles of ATP per mole of glucose formed. Thus cancer patients have been reported to have an increased oxidation of fat (Hyltander *et al*, 1991) and an increased rate of removal of infused lipids from the blood (Waterhouse and Nye, 1961). Increased utilisation of fatty acids as the preferred energy source has been observed even in the presence of high glucose concentrations (Waterhouse and Kemperman, 1971). This suggests that in the presence of certain tumours host tissues may increase their utilisation of fatty acids as an energy source. A number of studies have shown that such tumours elaborate lipid mobilising factors, which increase lipolysis in adipose tissue through the normal cyclic AMP-mediated pathway.

LMF has been extracted from the urine of patients with carcinoma of the stomach, rectum, pancreas, ovary and liver, where weight loss was established, but was absent from the urine of patients without weight loss, or from normal subjects (Todorov *et al*, 1998). This suggests that LMF may be involved in lipid depletion in cancer cachexia, although no measurements have been made on circulating levels of LMF. LMF of similar molecular weight and electrophoretic mobility was also isolated from the cachexia-inducing MAC16 murine tumour and is most likely involved in lipid depletion in this model (Beck and Tisdale, 1987), since there is no evidence for cytokine involvement (Mulligan *et al*, 1992a). The concentration of LMF which we have employed in the present study (8 μg per injection) was chosen based on the content of LMF in the MAC16 tumour (Todorov *et al*, 1998) and this concentration was previously found to be effective in depletion of adipose tissue in normal mice (Hirai *et al*, 1998). Maximum lipolytic activity was observed in mice bearing the MAC16 tumour when they had lost 15% of their body weight (Groundwater *et al*, 1990). At this point the animals had lost 40% of their body fat (Smith and Tisdale, 1993), which is close to the value (42%) obtained by the injection of normal NMRI mice with 8 μg LMF bi-daily (Hirai *et al*, 1998). Many of the effects of LMF in mice are similar to those produced by the MAC16 tumour. Thus both produce hypoglycaemia (Mcdevitt and Tisdale, 1992), lipid depletion (Beck and Tisdale, 1987) and an increased ^14^CO_2_ production from [^14^C] triolein (Mulligan *et al*, 1992b) indicative of an increase in lipid utilisation. Hypoglycaemia induced by LMF arises from an increased glucose consumption by the brain, heart, BAT and gastrocnemius muscle. The mechanism for the increased glucose consumption is not known, but appears to occur in a non-insulin-dependent manner (Islam-Ali and Tisdale, 2001a) and probably arises from an increased glucose transport and phosphorylation due to an increase in GLUT1 or GLUT3. LMF increases metabolic substrate utilisation *in vivo*. Thus overall glucose oxidation was increased by LMF, as measured by the formation of ^14^CO_2_ from D-[U-^14^C] glucose. LMF also produced an increase in oxidation of lipid, and the increased lipid accumulation by BAT and WAT after LMF administration may be due to rapid lipid turnover in these tissues. LMF has been shown not only to initiate lipolysis, but also to prime adipose tissue towards lipolytic stimuli by increasing Gαs expression with a reciprocal decrease in Gαi (Islam-Ali *et al*, 2001b). This together with the increase in UCP1 mRNA in BAT (Bing *et al*, 2002) after LMF suggests a role also in the disposal of excess lipid. Large amounts of micro-droplets of lipid have been shown to be deposited in hepatocytes after LMF administration (Bing *et al*, 2002), suggesting that lipid is mobilised at a faster rate than it can be metabolised in BAT, and that excess lipid is stored in the liver.

The results of this study confirm that LMF stimulates oxidative metabolism in whole animals, thus accounting for the lipid depletion (Hirai *et al*, 1998). The similarity between the effects of LMF on fuel disposal and that seen in cancer cachexia suggests that it may be responsible for the progressive lipid depletion.

## References

[bib1] BeckSATisdaleMJ1987 Production of lipolytic and proteolytic factors by a murine tumor-producing cachexia in the host Cancer Res 47 59195923 3311359

[bib2] BergMFrakerDLAlexanderHR1994 Characterization of differentiation factor/leukaemia inhibitory factor effect on lipoprotein lipase activity and mRNA in 3T3-L1 adipocytes Cytokine 6 425432 7948751 10.1016/1043-4666(94)90067-1

[bib3] BingCRussellSTBeckettEECollinsPTaylorSBarracloughRTisdaleMJWilliamsG2002 Expression of uncoupling proteins -1, -2 and -3 mRNA is induced by an adenocarcinoma-derived lipid-mobilizing factor Br J Cancer 86 612618 11870545 10.1038/sj.bjc.6600101 PMC2375279

[bib4] FearonKCH1992 The mechanisms and treatment of weight loss in cancer Proc Nutr Soc 51 251265 1438334 10.1079/pns19920036

[bib5] FerrePLeturgueABurnolA-PPenicaudLGiradJ1985 A method to quantify glucose utilization in vivo in skeletal muscle and white adipose tissue of the anaesthetized rat Biochem J 228 103110 3890836 10.1042/bj2280103 PMC1144958

[bib6] GroundwaterPBeckSABartonCAdamsonCFerrierINTisdaleMJ1990 Alteration of serum and urinary lipolytic activity with weight loss in cachectic cancer patients Br J Cancer 62 816821 2245173 10.1038/bjc.1990.384 PMC1971511

[bib7] HolroydeCPGabuzdaTGPutnamRCPaulPReichardGA1975 Altered glucose metabolism in metastatic carcinoma Cancer Res 35 37103714 1192429

[bib8] HiraiKHusseyHJBarberMDPriceSATisdaleMJ1998 Biological evaluation of a lipid-mobilizing factor isolated from the urine of cancer patients Cancer Res 58 23592365 9622075

[bib9] HyltanderADrottCKornerUSandstromRLundholmK1991 Elevated energy expenditure in cancer patients with solid tumours Eur J Cancer 27 915 1826450 10.1016/0277-5379(91)90050-n

[bib10] Islam-AliBSTisdaleMJ2001a Effect of a tumour-produced lipid-mobilizing factor on protein synthesis and degradation Br J Cancer 84 16481655 11401319 10.1054/bjoc.2001.1834 PMC2363694

[bib11] Islam-AliBKhanSPriceSATisdaleMJ2001b Modulation of adipocyte G-protein expression in cancer cachexia by a lipid-mobilizing factor Br J Cancer 85 758763 11531264 10.1054/bjoc.2001.1992 PMC2364135

[bib12] JenkinsABFurlerSMKraegenEW1986 2-Deoxy-D-glucose metabolism in individual tissues of the rat *in vivo* Int J Biochem 18 311318 3519306 10.1016/0020-711x(86)90036-4

[bib13] LacknerRAChallissRAJWestDNewsholmeAA1984 A problem in the radiochemical assay of glucose-6-phosphate in muscle Biochem J 218 649651 6324762 10.1042/bj2180649 PMC1153387

[bib14] LegaspiAJeevanandamMStarnesHFBrennanMF1987 Whole-body lipid and energy metabolism in the cancer patient Metabolism 36 958963 3657515 10.1016/0026-0495(87)90132-6

[bib15] MahonySMTisdaleMJ1990 Metabolic effects of tumour necrosis factor alpha in NMRI mice Br J Cancer 61 514519 2331437 10.1038/bjc.1990.116 PMC1971354

[bib16] McDevittTMTisdaleMJ1992 Tumour-associated hypoglycaemia in a murine cachexia model Br J Cancer 66 815820 1358167 10.1038/bjc.1992.366 PMC1977981

[bib17] MeszarosKBagbyGJLangCHSpitzerJJ1987a Increased uptake and phosphorylation of 2-deoxyglucose by skeletal muscles in endotoxin-treated rats Am J Physiol 253 E33E39 3300364 10.1152/ajpendo.1987.253.1.E33

[bib18] MeszarosKLangCHBagbyCJSpitzerJJ1987b Contribution of different organs to increased glucose consumption after endotoxin administration J Biol Chem 262 1096510970 3301848

[bib19] MulliganHDTisdaleMJ1991 Lipogenesis in tumour and host tissues in mice bearing colonic adenocarcinomas B J Cancer 63 719722 10.1038/bjc.1991.162 PMC1972399 1674876

[bib20] MulliganHDMahonySMRossJATisdaleMJ1992a Weight loss in a murine cachexia model is not associated with the cytokines tumour necrosis factor-α or interleukin-6 Cancer Lett 65 239243 1516039 10.1016/0304-3835(92)90238-q

[bib21] MulliganHDBeckSATisdaleMJ1992b Lipid metabolism in cancer cachexia Br J Cancer 66 5761 1637677 10.1038/bjc.1992.216 PMC1977893

[bib22] Oller do NascimentoCMWilliamsonDH1986 Evidence for conservation of dietary lipid in the rat during lactation and the immediate period after removal of the litter: Decreased oxidation of oral [1-^14^C]-triolein Biochem J 239 233236 3099779 10.1042/bj2390233 PMC1147266

[bib23] SmithKLTisdaleMJ1993 Increased protein degradation and decreased protein synthesis in skeletal muscle during cancer cachexia Br J Cancer 67 680685 8471425 10.1038/bjc.1993.126 PMC1968351

[bib24] StansbieDBrownseyRWCrettazMDentonRM1976 Acute effects *in vivo* of anti-insulin serum on rates of fatty acid synthesis and activities of acetyl-Coenzyme A carboxylase and pgruvate dehydrogenase in liver and epididymal adipose tissue of fed rats Biochem J 160 413416 12755 10.1042/bj1600413 PMC1164250

[bib25] ThompsonMPCooperSTParryBRTuckeyJA1993 Increased expression of the mRNA for the hormone-sensitive lipase in adipose tissue of cancer patients Biochim Biophys Acta 1180 236242 8422428 10.1016/0925-4439(93)90044-2

[bib26] TodorovPTMcDevittTMMeyerDJUeyamaHOhkuboITisdaleMJ1998 Purification and characterization of a tumor lipid-mobilizing factor Cancer Res 58 23532358 9622074

[bib27] WaterhouseCNyeWHR1961 Metabolic effects of infused triglycerides Metabolism 10 403414 13783352

[bib28] WaterhouseCKempermanJH1971 Carbohydrate metabolism in subjects with cancer Cancer Res 31 12731278 5286579

[bib29] WorkmanPTwentymanPBalkwillFBalmainAChaplinDDoubleJEmbletonJNewellDRaymondRStablesJStephensTWallaceJ1998 United Kingdom Co-ordinating Committee on Cancer Research (UKCCR)Guidelines for the welfare of animals in experimental neoplasia (second edition)Br J Cancer 77 110 10.1038/bjc.1998.1 PMC2151254 9459138

